# The value of CT findings combined with inflammatory indicators for preoperative differentiation of benign and malignant gallbladder polypoid lesions

**DOI:** 10.1186/s12957-023-02941-x

**Published:** 2023-02-18

**Authors:** Juan Zhang, Yuxian Wu, Yayuan Feng, Jiazhao Fu, Ningyang Jia

**Affiliations:** 1 Department of Radiology, Eastern Hepatobiliary Surgery Hospital, Third Affiliated Hospital of Naval Medical University, No.225 Changhai, Shanghai, 200433 China; 2 Department of Organ Transplantation, Changhai Hospital, First Affiliated Hospital of Naval Medical University, Shanghai, 200433 China

**Keywords:** Gallbladder polypoids, Computed tomography, Inflammatory indicator, Nomogram

## Abstract

**Background:**

The study aimed to explore the value of CT findings and inflammatory indicators in differentiating benign and malignant gallbladder polypoid lesions before surgery.

**Methods:**

The study comprised a total of 113 pathologically confirmed gallbladder polypoid lesions with a maximum diameter ≥ 1 cm (68 benign and 45 malignant), all of which were enhanced CT-scanned within 1 month before surgery. The CT findings and inflammatory indicators of the patients were analyzed by univariate and multivariate logistic regression analysis to identify independent predictors of gallbladder polypoid lesions, and then a nomogram distinguishing benign and malignant gallbladder polypoid lesions was developed by combining these characteristics. The receiver operating characteristic (ROC) curve and decision curve were plotted to assess the performance of the nomogram.

**Results:**

Base status of the lesion (*p* < 0.001), plain CT value (*p* < 0.001), neutrophil–lymphocyte ratio (NLR) (*p* = 0.041), and monocyte-lymphocyte ratio (MLR) (*p* = 0.022) were independent predictors of malignant polypoid lesions of the gallbladder. The nomogram model established by incorporating the above factors had good performance in differentiating and predicting benign and malignant gallbladder polypoid lesions (AUC = 0.964), with sensitivity and specificity of 82.4% and 97.8%, respectively. The DCA demonstrated the important clinical utility of our nomogram.

**Conclusion:**

CT findings combined with inflammatory indicators can effectively differentiate benign and malignant gallbladder polypoid lesions before surgery, which is valuable for clinical decision-making.

## Introduction

Gallbladder polypoid lesions (GPs) are clinically common gallbladder diseases, affecting about 5% of the adult population in Western countries [1, 2], but substantially higher in Eastern countries (7.4–9.9%) [3]. GPs appear on imaging as lesions that bulge from the gallbladder wall towards the lumen. Clinically, the majority of gallbladder polyps are benign, with only a few being malignant lesions [4]. Common benign GPs include cholesterol polyps, inflammatory polyps, and gallbladder adenoma, while gallbladder adenocarcinoma is the most common malignant GP. The treatment modalities for the two are very different, with clinical practice guidelines for the treatment of gallbladder cancer [5] stating that for early-stage gallbladder cancer, open surgery with peripheral lymph node dissection is the best treatment, whereas benign polypoid lesions can be treated conservatively or with laparoscopic surgery. Furthermore, their prognoses are drastically different, with malignant GPs having a poor prognosis, with a 5-year survival rate of only approximately 5% and a mean overall survival time of about 6 months [6]. Therefore, preoperative differentiation of benign and malignant GPs is critical for clinical treatment strategy selection and prognosis evaluation.

CT examinations have been increasingly used to evaluate gallbladder polypoid lesions [7–10]. But, the preoperative differentiation of benign and malignant GPs of the gallbladder remains challenging. To improve the accuracy of the prediction of benign and malignant GPs, several studies have developed multiparametric prediction models that include clinical features, imaging, and radiomics features [11–13]. Han et al. [12] established a nomogram for predicting benign and malignant polypoid lesions and showed that a model combining clinical features and CT-radiomics (AUC = 0.950) could provide a preoperative prediction of benign and malignant identification. However, feature determination and mapping of regions of interest for feature extraction are highly operator-dependent and unstable performance. Therefore, a convenient, fast, relatively stable method for preoperative discrimination of benign and malignant GPs is urgently needed.

In recent years, inflammation has got a lot of attention because it plays an important role in tumor growth by promoting tumor cell proliferation, invasion, and angiogenesis [14]. Neutrophil–lymphocyte ratio (NLR) [15–18], monocyte-lymphocyte ratio (MLR) [16, 19], platelet-lymphocyte ratio (PLR) [17, 18], and systemic inflammation response index (SIRI) [20], all of which represent the body’s inflammatory status, have been linked to the prognosis of gallbladder carcinoma in several prior studies. Chen [21] et al. reported that NLR combined with CA19-9 improved the preoperative diagnosis of gallbladder cancer; however, their study included all patients with gallbladder cancer and not only malignant GPs. To our knowledge, there have been no reports in which CT findings combined with inflammatory indicators to distinguish the nature of GPs. Therefore, the purpose of this study was to investigate the value of CT features combined with inflammatory indicators for preoperatively differentiating benign and malignant GPs.

## Material and methods

### Patients

**Fig. 1:**
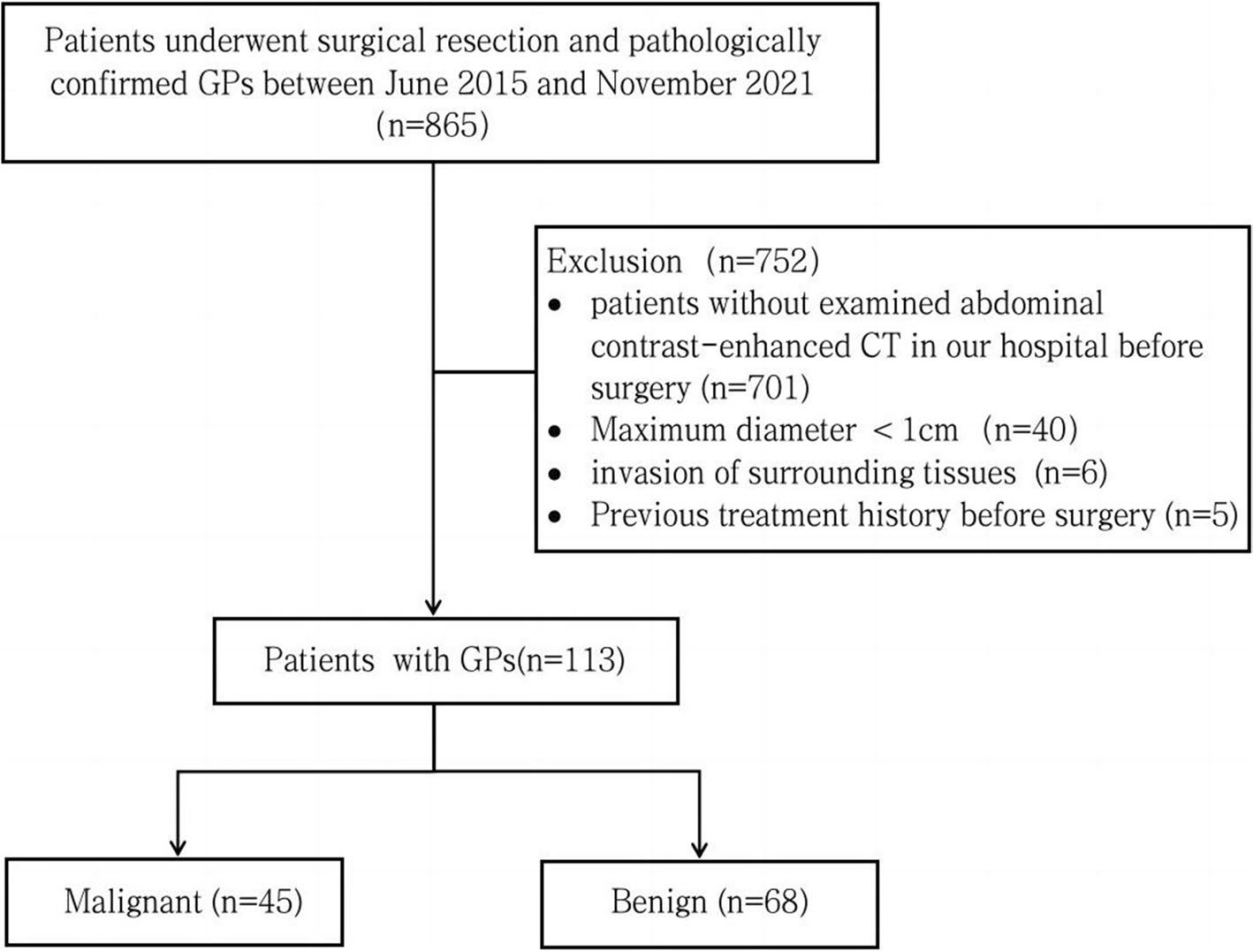
Flowchart of this study population

This retrospective study was approved by the Ethics Committee of Third Affiliated Hospital of Naval Medical University, and the requirement for informed patient consent was eliminated. Between January 2015 and December 2021, the researchers collected pathologically confirmed GPs with complete preoperative enhanced CT data, and 113 patients were eventually enrolled in the study, with 45 malignant GPs and 68 benign GPs. The inclusion criteria were as follows: (1) the largest diameter of the lesion was ≥ 1 cm; (2) the upper abdominal enhancement CT examination was performed in our hospital within 1 month before surgery; (3) complete pathological data were available after cholecystectomy. The following were the criteria for exclusion: (1) the lesion had invaded surrounding tissues, such as the liver and hepatic hilar lymph node; (2) patients underwent some operations and treatment before surgery including radiochemotherapy and percutaneous transhepatic gallbladder drainage; (3) the image was indistinct. The flow chart of patient registration is shown in Fig. 1.

### CT acquisition

The CT scan was performed on a GE Discovery 750HD multi-row spiral machine. The scanning parameters were as follows: tube voltage, 120 kV; reference tube current, 250–280 mAs; matrix, 512 × 512; pitch, 1.0; scan thickness and layer spacing, 5 mm. The patient fasted for at least 4 h before the CT scan, and the examination was performed in the traditional supine position, with an unenhanced scan first and subsequently an enhanced scan. Enhancement scans were performed using ioversol (HENGRUI MEDICAL, HR, China) ratio with a total injection volume of 1.5 ml/kg, injected through the elbow vein at a rate of 2.5–3.5 ml/s. Following the acquisition of unenhanced CT images, arterial phase, portal venous phase, and delayed phase scans were performed for 23–26 s, 50–60 s, and 120–160 s, respectively.

### Clinical data

The following clinical information was collected from the medical record system: (a) demographic characteristics (gender, age); (b) clinical symptoms: (absent or present); (c) BMI (weight/height^2^); (d) liver functional parameters (alanine aminotransferase [ALT], aspartate aminotransferase [AST], albumin [ALB], total bilirubin [TBIL], total bile acid [TBA]; (e) the following data were obtained from the neutrophil count, lymphocyte count, monocyte count, platelets: NLR (neutrophil to lymphocyte ratio), PLR (platelet to lymphocyte ratio), MLR (monocyte to lymphocyte ratio), SIRI (neutrophil × monocyte/lymphocyte); (f) tumor biomarkers (carcinoembryonic antigen [CEA], carbohydrate antigen 19–9 [CA19-9].

### Imaging feature

All CT scans were performed using the picture archiving and communication system (PACS; Pathspeed, Pathspeed, GE Medical Systems Integrated Imaging Solutions) and were independently evaluated by two radiologists imaging features. The two radiologists did not know the clinical indicators or pathological findings. A consensus was reached by joint discussion when there was disagreement between the two physicians. The main observations were the maximum length of the lesion; its location (divided into base, base + body, body, body + neck, and neck); the number of the lesion (single or multiple); the base status (the base width of GPs was as about Yamada’s classification: the angle between the protuberance of the GPs and the basement mucosa > 90° is referred to as the wide base, whereas the angle < 90°is defined narrow base [11]); gallbladder stones (absent or present); and lesions CT values in the plain and triphasic dynamic enhanced scan.

### Statistical analysis

All statistical analyses were performed using SPSS (version 26.0; IBM) and R software (Version 3.6.1). Student’s *t* test was used to compare continuous variables with a normal distribution, and the Mann–Whitney *U* test was used to analyze variables with an abnormal distribution. Categorical variables were compared using the *χ*^2^ test or Fisher’s exact test. NLR, PLR, LMR, MLR, and SIRI were converted to categorical variables based on receiver operating characteristic (ROC) curves. Univariate and multivariate logistic regression analyses were used to determine independent predictors of benign and malignant GPs. A two-sided *P* value < 0.05 was considered statistically significant. Based on the results from the multivariate logistic regression analysis, a nomogram model for differentiating benign and malignant GPs was developed, which underwent internal validation using the bootstrap method. Sensitivity, specificity, and area under the ROC curve of the nomogram were calculated. The accuracy and clinical utility of the nomogram were evaluated using decision curve analysis.

## Results

### Clinical characteristics

A total of 113 patients with benign and malignant GPs (malignant *n* = 45, benign *n* = 68) were enrolled in this study, of which approximately 73 (64.6%) were women and 40 (35.4%) were men. Age (*P* < 0.001), Lymphocyte (*P* = 0.016), NLR (*P* = 0.006), MLR (*P* < 0.001), SIRI (*P* = 0.002), ALB (*P* < 0.001), and CA19-9 (*P* = 0.008) were significantly different between the benign and malignant groups (*P* < 0.05), according to the results of the recorded clinical information. Other clinical indications did not differ significantly between the two groups (Table 1).

**Table 1:** Clinical characteristics of patients with GPs

Clinical parameters	Malignant (*n* = 45)	Benign (*n* = 68)	*p value*
Age			** < 0.001**
< 60	13 (28.89)	44 (64.71)	
≥ 60	32 (71.11)	24 (35.29)	
Gender			0.438
Male	14 (31.11)	26 (38.24)	
Female	31 (68.89)	42 (61.76)	
BMI			0.098
< 24	25 (55.56)	27 (39.71)	
≥ 24	20 (44.44)	41 (60.29)	
Symptoms			0.344
Yes	33 (73.33)	55 (80.88)	
No	12 (26.67)	13 (19.12)	
WBC	5.56 ± 1.74	6.01 ± 2.70	0.325
Neutrophil	4.52 ± 6.98	3.63 ± 2.54	0.337
Lymphocyte	1.62 ± 0.55	1.89 ± 0.62	**0.016**
Monocyte	0.36 ± 0.11	0.36 ± 0.16	0.755
PLT	201.47 ± 71.65	217.32 ± 61.55	0.212
NLR			**0.006**
≤ 1.45	6 (13.33)	25 (36.76)	
> 1.45	39 (86.67)	43 (63.24)	
MLR			** < 0.001**
≤ 0.24	24 (53.33)	57 (83.82)	
> 0.24	21 (46.67)	11 (16.18)	
PLR			0.087
≤ 145.69	30 (66.67)	55 (80.88)	
> 145.69	15 (33.33)	13 (19.12)	
SIRI			**0.002**
≤ 0.922	29 (64.44)	60 (88.24)	
> 0.922	16 (35.56)	8 (11.76)	
TBil	12.00 (9.50, 15.30)	12.10 (9.28, 16.02)	0.826
TBA	4.60 (2.60, 7.00)	3.90 (2.82, 5.30)	0.333
ALB	41.05 ± 4.59	43.72 ± 3.40	**0.001**
ALT	16.00 (13.00, 25.00)	15.50 (12.00, 24.00)	0.372
AST	17.00 (14.00, 20.00)	16.00 (14.00, 19.25)	0.275
CEA	2.10 (1.30, 3.10)	1.70 (1.10, 3.02)	0.249
CA19-9	12.00 (9.00, 18.90)	7.60 (4.35, 15.45)	**0.008**

The bold value means statistical significance

*WBC* White blood cell, *NLR* Neutrophil to lymphocyte ratio, *MLR* monocyte to lymphocyte ratio, *PLR* Platelet to lymphocyte ratio, SIRI = (neutrophil × monocyte)/lymphocyte, *TBil* Total bilirubin, *TBA* Total bile acid, *ALT* Alanine aminotransferase, *AST* Aspartate aminotransferase, *ALB* Albumin, *CEA* Carcinoembryonic antigen, *CA 19–9* Carbohydrate antigen 19–9

### CT imaging findings

**Fig. 2:**
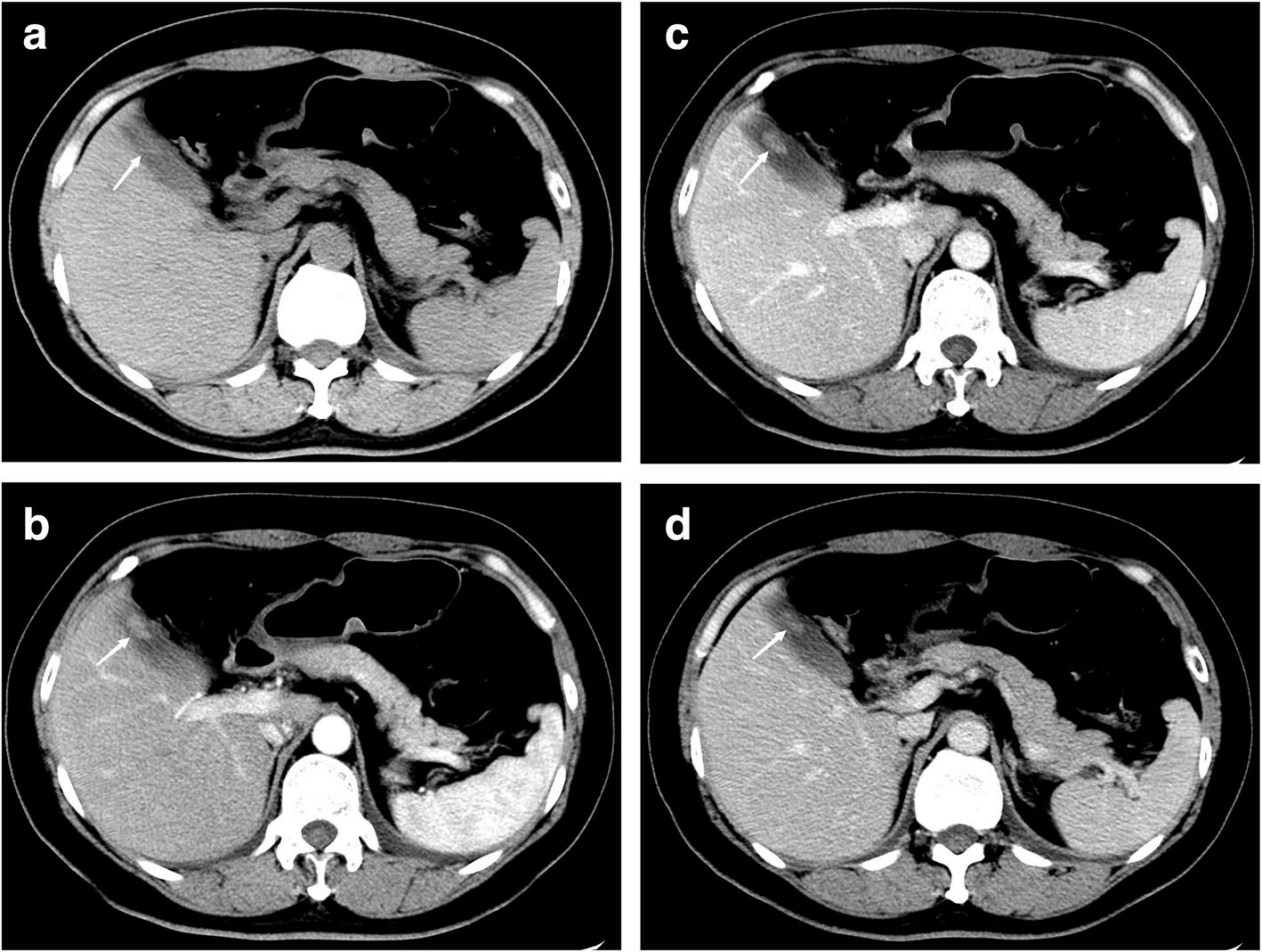
CT images of a 50-year-old woman with benign gallbladder polypoid lesion (gallbladder adenoma), which was narrow base. **a** Plain phase CT values was 36 HU, **b** artery phase CT values was 75 HU, **c** portal venous phase CT value was 74 HU, and **d** delayed phase CT value was 60 HU

**Fig. 3:**
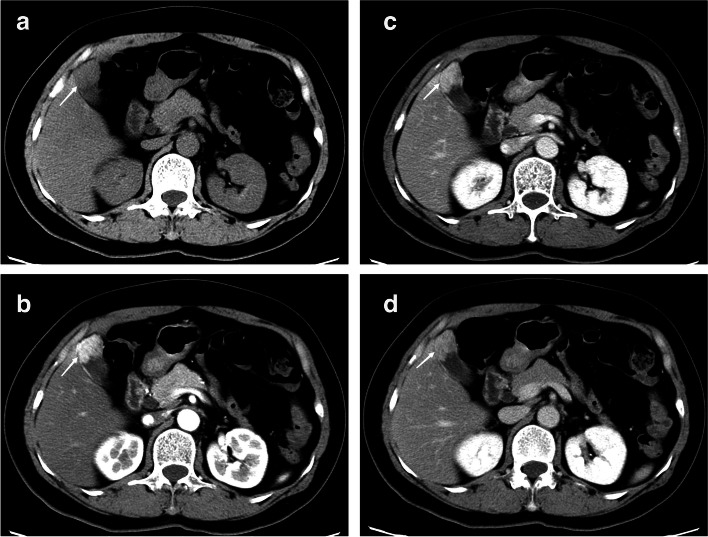
A 62-year-old women patient with gallbladder polypoid lesions (gallbladder carcinoma), which was wild base. **a** Plain phase CT values was 52 HU, **b** artery phase CT values was 102 HU, **c** portal venous phase CT value was 95 HU, and **d** delayed phase CT value was 87 HU

The maximal diameter of the lesions (*P* < 0.001), base status (*P* < 0.001), and the CT values of each phase (plain, arterial, portal, and delayed) (*P* < 0.001) were significantly different between the two groups among the recorded CT characteristics (Figs. 2 and 3). In this study, there were 96 single lesions (84.96%), of which about 60.4% and 39.6% were benign and malignant GPs, respectively; however, there was no significant difference in the number of lesions between the two groups. Furthermore, the location of the lesions and the presence of gallbladder stones were not related to the benign and malignant GPs (Table 2).

**Table 2:** Comparison of CT imaging features of GPs

Imaging parameters	Malignant (*n* = 45)	Benign (*n* = 69)	*p value*
Maximum diameter	2.40 (1.70, 3.10)	1.35 (1.10, 1.72)	** < 0.001**
Number			0.902
Single	38 (84.44)	58 (85.29)	
Multiple	7 (15.56)	10 (14.71)	
Location			0.099
Bottom	28 (62.22)	34 (50.00)	
Bottom + Body	1 ( 2.22)	8 (11.76)	
Body	7 (15.56)	19 (27.94)	
Body + Neck	2 ( 4.44)	2 ( 2.94)	
Neck	7 (15.56)	5 ( 7.35)	
Base			** < 0.001**
Narrow	7 (15.56)	59 (86.76)	
Wide	38 (84.44)	9 (13.24)	
Gallbladder stone			0.804
Absent	39 (86.67)	60 (88.24)	
Present	6 (13.33)	8 (11.76)	
CT value			
Plain phase CT value	49.39 ± 9.47	35.77 ± 11.38	** < 0.001**
Arterial phase CT value	84.72 ± 20.68	64.95 ± 17.25	** < 0.001**
Portal phase CT value	93.08 ± 17.59	71.33 ± 15.47	** < 0.001**
Delay phase CT value	86.67 ± 15.21	67.65 ± 14.29	** < 0.001**

The bold value means statistical significance

### Univariate and multivariate analyses

The following variables with *P* < 0.05 after univariate logistic regression analysis: age, NLR, MLR, SIRI, ALB, maximum diameter, base status, plain CT value, arterial phase CT value, portal phase CT value. Then, using stepwise backward multivariate logistic regression analysis of the above parameters showed that NLR (odds ratio [OR], 9.428; 95% confidence interval [CI], 1.095, 81.139; *P* = 0.046), MLR (OR, 7.295; 95% CI, 1.338, 39.777; *P* = 0.022), base status (OR, 71.516; 95% CI, 12.919, 395.883; *P* < 0.001), and plain CT value (OR, 1.158; 95% CI, 1.074, 1.249; *P* < 0.001) were independently associated with malignant GPs (Table 3).

**Table 3:** Univariate and multivariate analyses of risk factors for malignant GPs

Risk factor	Univariate analysis		Multivariate analysis	
	OR (95% CI)	*p value*	OR (95% CI)	*p* value
Age	4.513 (1.999, 10.187)	** < 0.001**		
NLR	3.779 (1.403, 10.179)	**0.009**	9.428 (1.095, 81.139)	**0.041**
MLR	4.534 (1.897, 10.839)	**0.001**	7.295 (1.338, 39.777)	**0.022**
SIRI	4.138 (1.588, 10.780)	**0.004**		
ALB	0.837 (0.746, 0.939)	**0.002**		
CA19-9	1.008 (0.988, 1.029)	0.434		
Maximum diameter	6.823 (3.252, 14.314)	** < 0.001**		
Base	35.587 (12.224, 103.601)	** < 0.001**	71.516 (12.919, 395.883)	** < 0.001**
Plain CT value	1.128 (1.075, 1.183)	** < 0.001**	1.158 (1.074, 1.249)	** < 0.001**
Arterial phase CT value	1.058 (1.032, 1.086)	** < 0.001**		
Portal phase CT value	1.081 (1.049, 1.115)	** < 0.001**		
Delay phase CT value	1.085 (1.051, 1.120)	** < 0.001**		

The bold value means statistical significance

*NLR* Neutrophil to lymphocyte ratio, *MLR* Monocyte to lymphocyte ratio, SIRI = (neutrophil × monocyte)/lymphocyte, *ALB* Albumin, *CA 19–9* Carbohydrate antigen 19–9

### Development and evaluation of the model

**Fig. 4:**
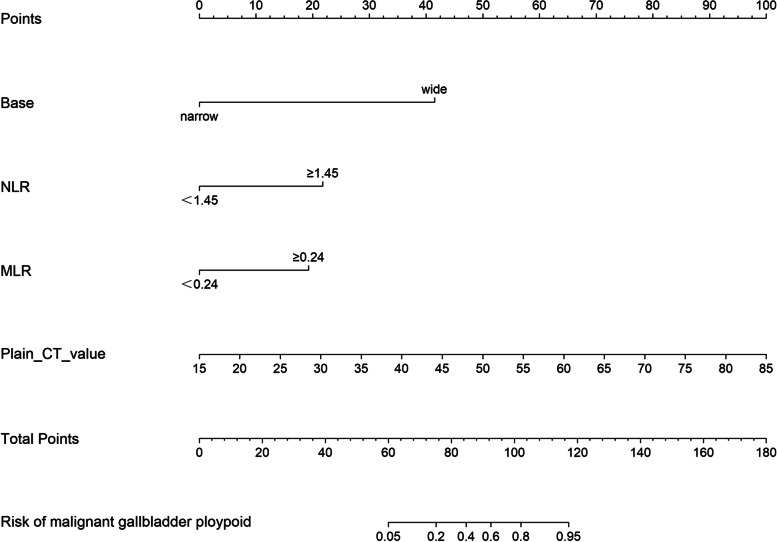
The nomogram diagnosed benign and malignant gallbladder polypoid lesions

**Fig. 5:**
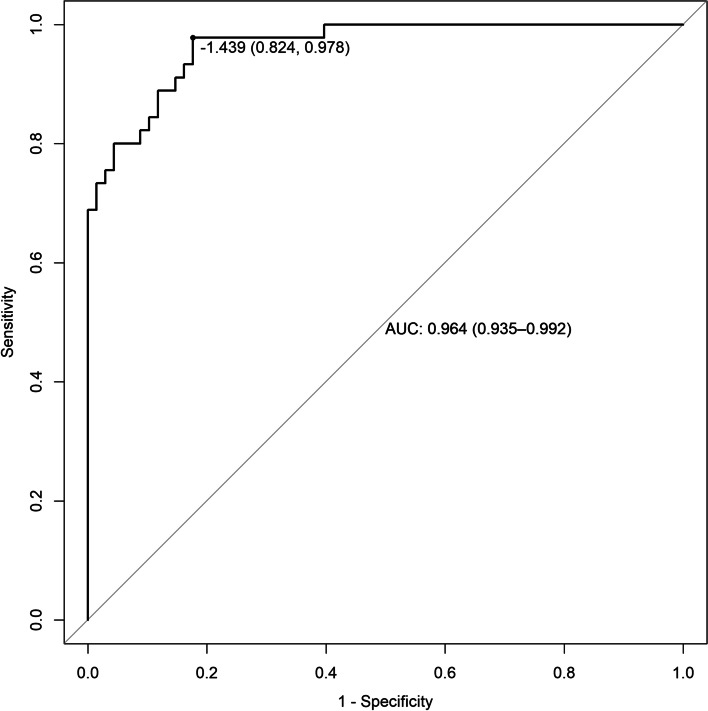
The model showed a higher area under the curve (AUC), sensitivity, and specificity

**Fig. 6:**
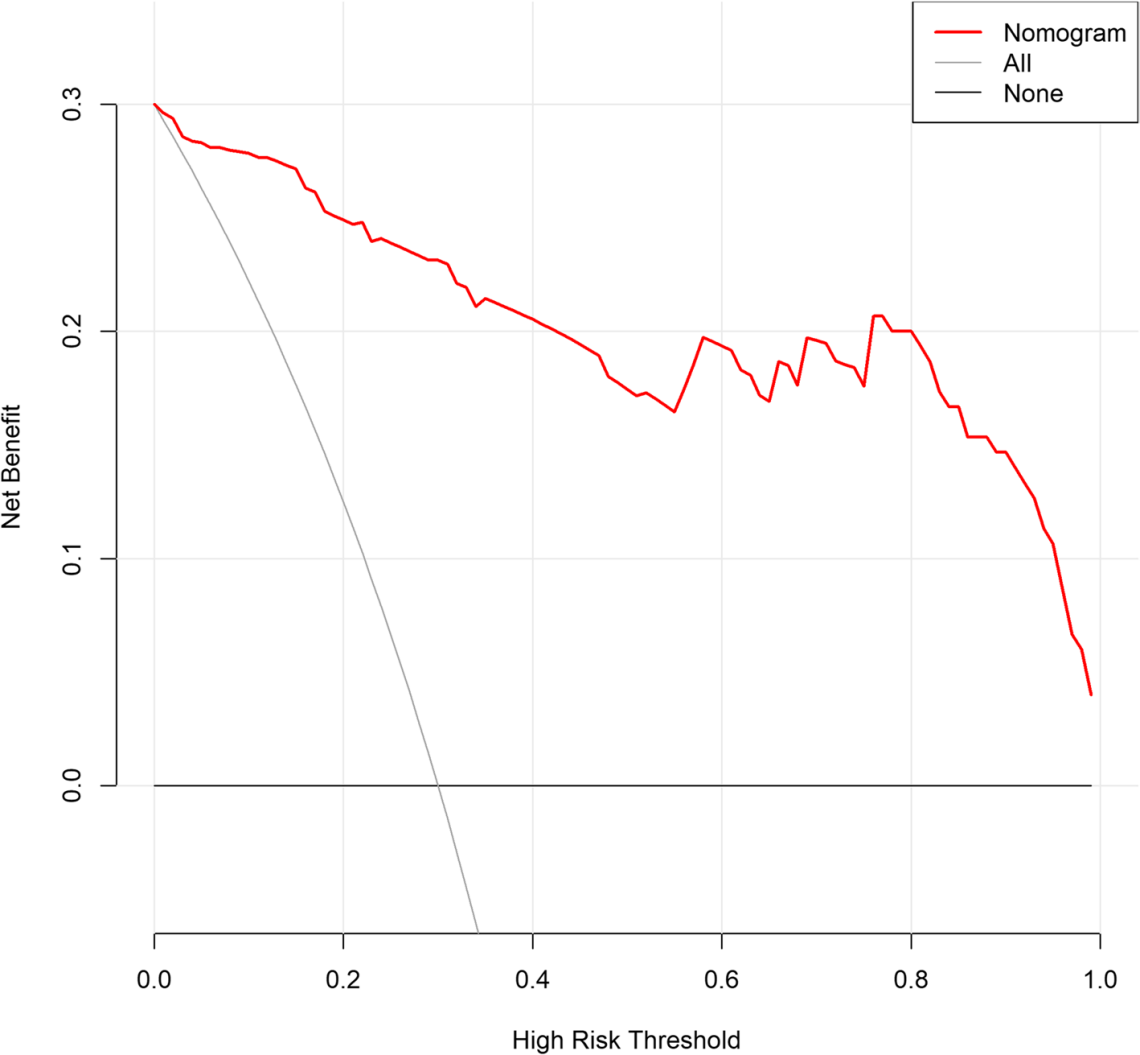
Decision curves demonstrated that a net benefit could be achieved when combining CT findings with inflammatory indicators

Based on the significant independent variables, a nomogram for predicting the probability of malignant GPs was developed (Fig. 4). The nomogram model was favorable with an area under the ROC curve of 0.964 (95% CI, 0.935–0.992), and the sensitivity and specificity were 82.4% and 97.8%, respectively, as shown in Fig. 5. The performance of the model was validated internally with an AUC of 0.951. The decision curve (Fig. 6) showed that the nomogram had a higher net benefit compared to the treat-all-patients strategy or the no-treatment strategy at different threshold probabilities.

## Discussion

The emphasis of this investigation was on GPs with a maximum diameter of ≥ 1 cm, and GPs with a diameter of ≤ 1 cm are usually managed by observational follow-up in clinical practice due to their low risk of malignancy [22]. Because the treatment and prognosis of benign and malignant gallbladder polypoid lesions are so extremely different, identifying the nature of GPs before surgery is crucial. In this study, we found that NLR (*P* = 0.041), MLR (*P* = 0.022), the base status of the lesion (*P* < 0.001), and plain CT value (*P* < 0.001) were independent risk factors for GPs, and the nomogram model based on this result could effectively distinguish and predict benign and malignant GPs ≥ 1 cm (AUC = 0.964). In addition, the model demonstrates better accuracy, which can provide important information for medical decision-making.

Ultrasound is the preferred method for diagnosing gallbladder polypoid lesions; however, it can be difficult to distinguish between benign and malignant GPs. CT is also often used to examine gallbladder disease because contrast enhancement allows the lesion to be distinguished from the surrounding tissue and aids in the diagnosis of gallbladder disease. The base appearance was found to be an independent predictor of benign and malignant GPs in this analysis, which is consistent with prior studies [11, 12]. The size of GPs is one of the most important indications of the nature of the tumor and the need for surgery in clinical practice. However, the research found that approximately 83% of GPs that underwent surgery (≥ 1 cm) were benign [23], indicating that assessing the nature of GPs solely based on the size of the lesion is not entirely reliable [24]. It was similarly shown in the present study that although the maximum diameter of the lesion was associated with benign and malignant GPs, it was not an independent predictor.

CT values at all phases were associated with malignant GPs in our study, yet only the plain CT value was found to be an independent predictor. This is consistent with the findings of a recent study [25], which demonstrated that plain CT value was useful for distinguishing gallbladder carcinoma from cholesterol polyps, whereas a previous study [7] concluded that plain CT value, delayed phase CT value, and portal phase–delayed phase CT value were all capable of detecting malignant GPs (*P* < 0.05). These differences could be attributable to sample bias or sample size disparities.

On the other hand, we have found some inflammatory indicators to be of diagnostic value in distinguishing benign and malignant GPs. Previously, researchers have concentrated more on the prognostic relationship between NLR and gallbladder carcinoma, in contrast to the limited studies focusing on NLR and preoperative diagnosis. The role of NLR has been investigated in several retrospective studies and was significantly associated with poor prognosis in GBC patients undergoing radical operation [16, 17]. Besides, NLR was also shown to be associated with poor outcomes in patients with metastatic GBC [26]. In this investigation, we discovered that patients with malignant GPs had considerably greater levels of NLR than patients with benign GPs, which is consistent with prior results. Researchers have suggested that increased neutrophil levels affect the production of the tumor microenvironment by inducing various cytokines and chemokines, which in turn accelerate tumor cell proliferation and metastasis [27–29]. On the other hand, lymphocytes have been found to exist at lower levels in patients with malignant tumors because of their ability to provide immune surveillance and inhibition of tumor cell maturation [30, 31]. And in this current investigation, lymphocytes were also significantly lower in malignant GPs. MLR is also an independent predictor of benign and malignant GPs, according to our findings. Choi et al. analyzed the outcomes of 178 patients with advanced gallbladder cancer and observed that MLR predicts independent survival in gallbladder cancer patients undergoing chemotherapy [19]. Moreover, Tao et al. have shown that MLR was found to have an inverse relationship with survival in GBC with hepatic involvement [16]. Due to the complexity of inflammatory indicators, the mechanism of MLR has remained unclear. Tumor-associated macrophages (TAMs) may play a role, which is the main component of infiltration in many tumor stroma. Published studies have reported that it has been speculated that TAMs can stimulate tumor cell proliferation, promote angiogenesis, and enhance invasion and metastasis [32]. Furthermore, high levels of SIRI have been related to a poor prognosis after gallbladder carcinoma surgery [20], whereas SIRI was linked to benign and malignant GPs in this study, but was not an independent risk factor.

We considered the constructed nomogram to be a useful and convenient method for preoperative differential and prediction of benign and malignant GPs due to the objective and precisely measurable acquisition of inflammatory indicators and the convenience and availability of CT examinations for preoperative examination. The model has an AUC of 0.964, indicating more diagnostic efficiency than prior investigations (AUCs of 0.856 [7], 0.875 [8], and 0.79 [9]). The use of this model may improve the preoperative diagnosis of this group of patients and provide a valuable reference for the choice of surgical approach and the assessment of prognosis.

Despite the more satisfactory findings, this study has several limitations. First, this was a retrospective study with a small number of cases, which might lead to bias. Second, the study enrolled the lesions which were maximum diameter ≥ 1 cm in size, which limits the applicability of the model results. Finally, because this is single-center research, more multicenter and large sample studies are required for external validation.

## Conclusion

In conclusion, this study presents a novel model based on CT findings and inflammatory indicators for the effective preoperative differentiation and prediction of benign and malignant GPs, which could guide the preoperative decision-making of malignant GPs ≥ 1 cm in size. In addition, it still needs to find more evidence and be confirmed by further clinical and multicenter studies.

## Data Availability

The datasets used or analyzed during the current study are available from the corresponding author on reasonable request.
